# A Pilot Study of Nutritional Status Prior to Bariatric Surgery in South China

**DOI:** 10.3389/fnut.2021.697695

**Published:** 2021-07-12

**Authors:** Linli Sun, Chunxia Wang, Wei Sun, Chunjiang Wang

**Affiliations:** ^1^Department of General Surgery, The Third Xiangya Hospital, Central South University, Changsha, China; ^2^Yinan County People's Hospital, Linyi, China; ^3^Department of Pharmacy, Third Xiangya Hospital, Central South University, Changsha, China

**Keywords:** obesity, bariatric surgery, sleeve gastrectomy, nutrient deficiency, micronutrient

## Abstract

**Objective:** The was a pilot study to assess the biochemical and historical information about bariatric patients before undergoing the surgery in the aim of identifying nutritional deficiencies and their prevalence from 2015 to 2020.

**Methods:** Clinical data of 247 patients (105 males and 142 females) were included. Vitamins, trace elements, electrolytes, albumin, globulin, hemoglobin, folate, ferritin, microalbuminuria (MAU), and parathyroid hormone (PTH) levels were determined to explore the nutritional status according to gender, age, high body mass index (BMI), and waist circumstance (WC).

**Results:** The mean age, mean BMI, and mean WC of the candidates were 32.95 ± 10.46 years, 38.01 ± 7.11 kg/m^2^, and 117.04 ± 16.18 cm, respectively. The prevalence of preoperative nutritional deficiencies was 76.88% for 25 (OH) vitamin D, 19.84% for globulin, 11.74% for albumin, 11.02% for sodium, 8.33% for folic acid, 10.48% (male) and 6.34% (female) for chloride, 4.05% for calcium, 3.07% (male) and 0.70%(female) for ferritin, 11.90% for elevated PTH, and 44.96% for MAU. Males exhibited increased prevalence of globulin and MAU relative to females (*P* < 0.05). Older groups are more likely to exhibit albumin deficiency (*P* = 0.007), globulin deficiency (*P* = 0.003), and zinc deficiency (*P* = 0.015). In addition, 25 (OH) D deficiency and albumin deficiency were more common in patients with BMI ≥ 47.5 kg/m^2^ (*P* = 0.049 and 0.015, respectively). Wider WC (≥150 cm) exhibited higher rates of albumin deficiencies (*P* = 0.011).

**Conclusion:** Electrolyte and nutritional deficiencies were common in patients prior to bariatric surgery in South China. Routine evaluation of electrolyte and nutritional levels should be carried out in this population.

## Introduction

Obesity is a global epidemic affecting populations globally and is closely related to hypertension, type 2 diabetes mellitus (T2DM), cardiovascular disease, chronic kidney disease, various cancers, a series of skeletal and muscular diseases, and all-cause mortality (1). The prevalence of obesity in China increased from 5.7% in the 2010 survey to 6.3% in the 2017 survey (2). Obesity and obesity-related complications also account for high medical costs and impose a large economic burden on the individual, families and nations (3). High body mass index (BMI) ranked the fourth among the leading risk factors in the Global Burden of Disease analysis and accounted for 4.72 million deaths and 148 million disability-adjusted life-years from 1990 to 2017 (4).

Bariatric surgery is presently considered the most effective approach to achieve greater weight loss and improve the quality of life, but also improve co-morbidities. Despite multiple clinical benefits, a number of surgical and gastrointestinal complications may occur after surgery (5). Among the possible complications, electrolyte and nutritional deficiencies are well-recognized long-term complications of bariatric surgery (6). Several recently published studies have reported that a considerable number of obese patients suffered from nutritional deficiencies prior to bariatric surgery (7–9). Although, nutritional deficiencies have not been identified as absolute contraindications for bariatric metabolic surgery, current guidelines strongly recommend correcting trace element deficiencies prior to surgery (10). These studies are mainly from Western countries, and a few are from Asian countries. Although, electrolyte/nutrient deficiencies have been reported, there are still few studies on the Chinese population. In addition, various dietary habits and lifestyles may lead to different nutrient statuses between different countries (11). China has its own characteristics compared with other countries. Chinese individuals have a lower BMI, wider waist circumference (WC), and more abdominal adiposity than white individuals (12). However, the link between electrolyte/nutrient data and patient demographic data (e.g., WC, age) in South China has not been well-elucidated.

In view of this, other research data may not be applicable to obese patients in South China. The purpose of this study is to investigate the prevalence of electrolyte/nutrition deficiencies among Chinese bariatric surgery candidates and to explore their relationship with patient demographic data and anthropometric characteristics.

## Methods

### Survey Participants

This study is a retrospective analysis of patients who were admitted to the Third Xiangya Hospital undergoing bariatric surgery from January 2015 to December 2020. According to the characteristics of the Chinese population, the World Health Organization (WHO) classifies BMI = 27.5 kg/m^2^ as general obesity (13). Inclusion criteria were patients aged 18–65 years with BMI ≥ 32.5 or 27.5 kg/m^2^ with ≥ 2 metabolic syndrome components or comorbidities that are unable to be controlled after lifestyle changes and medical treatment. The patients suffering from drug abuse, alcohol addiction, or uncontrollable mental illness, chronic renal failure, or who were taking multivitamin supplements or mineral supplements were excluded.

### Data Collection

Nutrient deficiencies were diagnosed by blood analysis. Surgery candidates draw fasting blood samples from peripheral veins after an overnight fast prior to surgery, which were then analyzed by the clinical laboratory of our hospital. Demographic data were collected on gender, age, WC, height, weight, HbA1C, and medical history. Related nutrients [hemoglobin, albumin, globulin, folate, vitamins, calcium, phosphorus, iron, copper, zinc, lead, cadmium, magnesium, ferritin, phosphorus, parathyroid hormone (PTH), and 25-OH-vitamin D] and some electrolytes (potassium, calcium, sodium and chloride) were collected.

### Definitions

Nutrient deficiencies were diagnosed by blood analysis. Laboratory values were regarded as deficient when they did not meet the reference values determined by our clinical laboratory. Microalbuminuria (MAU) was defined as clinical rise in the urinary albumin excretion >20 mg/L. BMI was calculated as the weight in kilograms divided by height in meters squared. To explore the associations between nutritional status and different physical state, we divided patients into several subgroups based on gender (male and female), age (18–29, 30–39, 40–49, and ≥50 years), BMI (27.5–32.5, 32.5–37.5, 37.5–42.5, 42.5–47.5, and ≥47.5 kg/m^2^), and WC (90–110, 110–130, 130–150, and ≥150 cm).

### Statistical Analysis

Statistical analysis was performed using SPSS 21.0 (Statistical Product and Service Solutions version 21.0) software for Windows. Continuous data were presented as the mean ± standard deviation, and count data were expressed as percentage (%). Subgroups were compared using the Chi-square (χ^2^) or Fisher's exact test for nutritional deficiencies. A multivariable logistic regression analysis was conducted to evaluate the associated factors for nutrient levels. *P* < 0.05 was considered to be statistically significant.

## Results

The clinical characteristics were shown in Table 1. A total of 247 patients (105 males and 142 females) with an average age of 32.95 ± 10.46 years were included. The mean BMI and WC were 38.01 ± 7.11 kg/m^2^ and 117.04 ± 16.18 cm, respectively. The prevalence of hypertension and diabetes was 57.49 and 38.46%, respectively. The prevalence of these conditions in men was higher than that in women (*P* < 0.05). Hypertriglyceridemia and hypercholesterolemia were found in 42.51 and 16.60% of the patients, respectively.

**Table 1:** The baseline clinical characteristics of study participants before bariatric surgery.

**Parameter**	**Total**	**Male**	**Female**	***P*-value**
No.	247	105 (42.51%)	142 (57.49%)	
Age (years)	32.95 ± 10.46	33.94 ± 10.81	32.22 ± 10.20	0.202
Weight (kg)	106.50 ± 24.40	117.14 ± 25.98	98.45 ± 19.91	0.000^*^
Height (cm)	166.67 ± 7.62	172.83 ± 5.79	162.05 ± 5.22	0.000^*^
BMI (kg/m^2^)	38.01 ± 7.11	39.25 ± 8.24	37.09 ± 6.20	0.020^*^
A1C (%)	7.17 ± 1.87	7.32 ± 1.92	6.64 ± 1.72	0.009^*^
WC (cm)	117.04 ± 16.18	123.60 ± 17.04	112.45 ± 13.95	0.000^*^
Medical history				
Hypertension	57.49%	66.67%	50.70%	0.012^*^
Diabetes	38.46%	51.43%	28.87%	0.000^*^
GERD	13.77%	16.19%	11.97%	0.341
Hypercholesterolemia	16.60%	20.95%	13.38%	0.114
Hypertriglyceridemia	42.51%	47.62%	38.73%	0.163
Fatty liver	92.71%	90.48%	94.37%	0.245
OSAS	56.68%	63.81%	51.41%	0.052
Hp (+)	22.67%	26.67%	19.72%	0.197

*BMI, body mass index; HbA1C, A1C; WC, waist circumstance; GERD, gastroesophageal reflux disease; OSAS, obstructive sleep apnea syndrome; HP, Helicobacter pylori*.

* *p <0.05*.

Patients' nutritional status prior to bariatric surgery was shown in Table 2. A total of 76.88% of the patients had a deficiencies in 25 (OH) vitamin D, 19.84% in globulin, 11.74% in albumin, 11.02% in sodium, 8.33% in folic acid, 7.69% in chloride, 4.05% in calcium, and 2.36% in ferritin. In addition, 11.90% exhibited elevated levels of PTH, and 44.96% exhibited MAU. Serum hemoglobin deficit appeared more prevalent in women than men (6.33 vs. 3.73%), while ferritin and chloride deficiency were more common in men (10.48 vs. 6.34%), and (3.07 vs. 0.70%), respectively. The lead level in male patients (36.26 ± 20.76 μg/L) was lower than that in female patients (29.18 ± 12.07 μg/L*, P* < 0.05), but they were all within the normal range. None of the patients exhibited deficiencies in vitamins (vitamin B2, vitamin B9, vitamin B12, vitamin C, vitamin E, and vitamin B6) and trace elements (iron, cadmium and magnesium).

**Table 2:** Biochemical parameters and comparison between male and female prior to bariatric surgery.

**Parameter**	**Reference values**	**Prevalence**	**Total**	**Male**	**Female**	***P*-value**
Albumin	40–55 g/L	11.74%	44.53 ± 4.68	45.50 ± 4.52	44.70 ± 3.77	0.130
Globulin	25–32 g/L	19.84%	28.09 ± 4.20	27.10 ± 3.87	28.39 ± 3.62	0.008^*^
Hemoglobin	Male: 130–170 g/L	3.73%	146.92 ± 16.33	156.67 ± 14.46	137.03 ± 11.77	0.000^*^
	Female: 120–150 g/L	6.33%				
25 (OH) D	20~100 ng/ml	76.88%	15.75 ± 7.30	15.56 ± 6.61	15.90 ± 7.82	0.745
Vitamin A	1.7–5.1 μmol/L	1.61%	2.66 ± 0.55	2.60 ± 0.50	2.70 ± 0.57	0.475
Vitamin B1	70–180 nmol/L	0.81%	96.91 ± 6.53	96.16 ± 6.51	96.21 ± 11.90	0.978
Vitamin B2	6–15 μg/L	0.00%	8.89 ± 1.39	8.29 ± 1.22	8.09 ± 1.48	0.449
Vitamin B9	6.8–156 nmol/L	0.00%	25.43 ± 13.36	25.12 ± 14.74	25.61 ± 12.47	0.845
Vitamin B12	170–900 pg/ml	0.00%	292.86 ± 71.09	296.87 ± 71.29	290.49 ± 70.87	0.633
Vitamin C	17–56.2 μmol/L	0.00%	26.44 ± 4.27	26.89 ± 4.42	26.18 ± 4.16	0.376
Vitamin E	5–12 μg/ml	0.00%	7.81 ± 1.11	7.94 ± 1.16	7.73 ± 1.08	0.321
Vitamin B6	41.8–132.3 nmol/L	0.00%	55.54 ± 10.11	55.28 ± 8.94	55.70 ± 10.73	0.827
Iron	Female: 9–27μmol/L	3.16%	8.29 ± 1.00	8.25 ± 0.86	8.25 ± 1.05	0.984
	Male: 11–30μmol/L					
Copper	11.8–39.3 μmol/L	2.34%	18.41 ± 5.09	19.53 ± 5.38	17.76 ± 4.80	0.060
Zinc	76.5–170 μmol/L	10.16%	87.81 ± 13.93	87.00 ± 11.54	89.26 ± 12.54	0.317
Lead	1.0–200.0 μg/L	0.00%	31.66 ± 16.27	36.26 ± 20.76	29.18 ± 12.07	0.017^*^
Cadmium	0–5 μg/L	0.00%	1.18 ± 1.05	1.33 ± 1.27	1.10 ± 0.89	0.281
Magnesium	1.12–2.06 mmol/L	0.00%	1.51 ± 1.00	1.42 ± 0.17	1.43 ± 0.14	0.801
Sodium	137–147 mmol/L	11.02%	140.89 ± 2.36	141.06 ± 2.44	140.77 ± 2.29	0.334
Potassium	3.5–5.3 mmol/L	1.62%	4.11 ± 0.32	4.14 ± 0.37	4.09 ± 0.28	0.246
Calcium^#^	2.2–2.7 mmol/L	4.05%	2.4 ± 0.14	2.41 ± 0.15	2.39 ± 0.13	0.216
Chloride	99–110 mmol/L	10.48%	103.4 ± 3.04	102.77 ± 2.92	103.94 ± 3.03	0.003^*^
		6.34%				
Phosphorus	0.85–1.51 mmol/L	0.00%	1.31 ± 0.35	1.42 ± 0.41	1.12 ± 0.16	0.227
Ferritin	Male 30–400 ng/ml	3.07%	224.35 ± 221.29	314.74 ± 251.54	126.55 ± 122.85	0.000^*^
	Female 13–150 ng/ml	0.70%				
Folate	4.6–34.8 ng/ml	8.33%	12.15 ± 5.44	12.06 ± 5.17	11.95 ± 5.38	0.940
PTH	15–65 ng/ml	11.90%	42.80 ± 20.34	44.31 ± 22.89	40.36 ± 15.00	0.553
MAU	0–20 mg/L	44.96%	68.62 ± 155.18	148.83 ± 264.17	29.88 ± 52.06	0.001^*^

*25 (OH) D, 25 hydroxyvitamin D; PTH, parathyroid hormone; MAU, microalbuminuria*.

# *Calcium levels were corrected by serum albumin*.

* *P <0.05*.

Different nutritional statuses were assessed before bariatric surgery according to gender, age, BMI, and WC (Table 3). Obvious gender differences were noted in globulin deficiency (28.57% in males and 13.38% in females, *P* = 0.003), sodium deficiency (0.95% in males and 7.04% in females, *P* = 0.022), MAU (59.65% in males and 33.33% in females, *P* = 0.003). Age group analyses revealed that older groups (≥50) were more likely to exhibit albumin deficiency (χ^2^ = 11.431, *P* = 0.007), globulin deficiency (χ^2^ = 13.833, *P* = 0.003), and zinc deficiency (χ^2^ = 9.450, *P* = 0.015). Patients with different BMIs had different albumin deficiency (χ^2^ = 11.634, *P* = 0.015) and 25 (OH) D (χ^2^ = 9.532, *P* = 0.049) deficiency rates. The albumin deficiency rate (34.62%) was highest in the BMI ≥ 47.5 kg/m^2^ group, and the 25 (OH) D deficiency rate (84.78%) was highest in the 37.5–42.5 kg/m^2^ BMI group. In terms of WC classifications, participants with wider WC (≥ 130 cm) had higher rates of deficiencies for albumin (χ^2^ = 10.348, *P* = 0.011) and sodium (χ^2^ = 7.170, *P* = 0.049).

**Table 3:** Associations between nutritional deficiencies and different physical states.

**Parameter**	**Albumin**	**Globulin**	**25 (OH) D**	**MAU**	**Folate**	**Zinc**	**Sodium**	**Chloride**	**Calcium**
Gender
Male (%)	13/105 (12.38)	30/105 (28.57)	68/91 (74.73)	34/57 (59.65)	2/35 (5.71)	5/47 (10.64)	1/105 (0.95)	11/105 (10.48)	5/105 (4.76)
Female (%)	16/142 (11.26)	19/142 (13.38)	85/108 (78.70)	24/72 (33.33)	3/25 (12)	7/93 (7.53)	10/142 (7.04)	9/142 (6.34)	5/142 (3.52)
*p*	0.788	0.003^*^	0.507	0.003^*^	0.385	0.535	0.022^*^	0.239	0.625
χ^2^	0.072	8.760	0.440	8.903	0.156	0.091	3.927	1.389	0.026
Age (years)
18–29 (%)	12/100 (12.00)	12/100 (12.00)	66/82 (80.49)	26/53 (49.06)	3/17 (17.66)	5/55 (9.09)	6/100 (6.00)	8/100 (8.00)	3/100 (3.00)
30–39 (%)	5/91 (5.49)	20/91 (21.98)	55/68 (94.83)	19/43 (44.19)	1/20 (5.00)	3/50 (6.00)	4/91 (4.40)	6/91 (6.59)	2/91 (2.20)
40–49 (%)	5/36 (13.89)	7/36 (19.44)	19/32 (59.38)	10/22 (45.45)	1/12 (8.33)	1/18 (5.56)	3/36 (8.33)	3/36 (8.33)	3/36 (8.33)
≥50	7/20 (35.00)	10/20 (50.00)	13/18 (72.22)	4/11 (36.363)	0/11 (0)	3/5 (60)	1/20 (5)	2/20 (10.00)	2/20 (10.00)
*p*	0.007^*^	0.003^*^	0.087	0.881	0.475	0.015^*^	0.821	0.885	0.149
χ^2^	11.431	13.833	6.488	0.667	2.606	9.450	1.083	0.717	4.792
BMI (kg/m^2^)
27.5–32.5 (%)	7/63 (11.11)	16/63 (25.40)	35/49 (71.43)	11/38 (28.95)	0/20 (0.00)	1/27 (3.70)	2/63 (3.17)	5/63 (7.94)	6/63 (9.52)
32.5–37.5 (%)	6/70 (8.57)	16/70 (22.886)	39/53 (73.58)	18/30 (60.00)	1/13 (7.69)	7/46 (15.22)	2/70 (2.86)	6/70 (8.57)	1/70 (1.43)
37.5– 42.5 (%)	4/58 (6.90)	7/58 (12.07)	39/46 (84.78)	16/36 (44.44)	1/14 (7.14)	3/26 (11.54)	6/58 (10.34)	5/58 (8.62)	1/58 (1.72)
42.5–47.5 (%)	3/30 (10.00)	5/30 (16.67)	20/26 (76.92)	6/11 (54.55)	2/8 (25.00)	1/15 (6.67)	2/30 (6.67)	2/30 (6.67)	2/30 (6.67)
≥47.5 (%)	9/26 (34.62)	5/26 (19.23)	20/25 (80.00)	7/13 (53.85)	1/5 (20.00)	0/14 (0.00)	2/26 (7.69)	1/26 (3.85)	0/26 (0.00)
*p*	0.015^*^	0.403	0.049^*^	0.105	0.101	0.403	0.314	0.982	0.098
χ^2^	11.634	4.023	9.532	7.605	5.877	3.718	4.407	0.626	6.812
WC (cm)
90–110 (%)	4/48 (4.55)	20/88 (22.73)	49/66 (74.24)	21/48 (43.75)	1/20 (5.00)	2/5 (40.00)	4/88 (4.55)	4/88 (4.55)	2/88 (2.27)
110–130 (%)	14/97 (14.43)	16/97 (6.4)	62/79 (78.48)	26/50 (52.00)	3/25 (12.00)	7/5 (12.96)	5/97 (5.15)	11/97 (11.34)	7/97 (7.22)
130–150 (%)	7/34 (20.59)	7/34 (20.59)	23/32 (71.88)	5/16 (31.25)	0/9 (0.00)	0/13 (0.00)	6/34 (17.65)	1/34 (2.94)	0/34 (0.00)
≥150 (%)	2/7 (28.57)	0/7 (0.00)	6/7 (85.71)	3/3 (100.00)	0/1 (0.00)	0/4 (0.00)	1/7 (14.29)	0/7 (0.00)	0/7 (0.00)
*P*	0.011^*^	0.492	0.799	0.003^*^	0.940	0.133	0.049^*^	0.262	0.240
χ^2^	10.348	2.378	1.041	13.432	0.504	4.821	7.170	3.734	3.765

*25 (OH) D, 25 hydroxyvitamin D; MAU, microalbuminuria; BMI, body mass index; WC, waist circumstance; χ^2^, Chi-square*.

* *P <0.05*.

### Disease Factors Related to Nutrients

A multivariable logistic regression analysis was performed to evaluate the the impact of related concomitant diseases on 25 (OH) D, MAU and PTH (Table 4). The results showed that hypertension, diabetes, GERD, and fatty liver were significantly correlated with 25 (OH) D deficiency and MAU; Diabetes, hypercholesterolemia, hypertriglyceridemia are significantly associated with elevated PTH.

**Table 4:** Multivariable logistic regression analysis of factors associated with 25 (OH) D, MAU. and PTH.

**Characteristic**	**25 (OH) D**		**MAU**		**PTH**	
	**OR (95% CI)**	***P***	**OR (95% CI)**	***P***	**OR (95% CI)**	***P***
Hypertension	51.128 (21.170,352.543)	0.000^*^	46.731 (31.734,278.945)	0.000^*^	0.632 (0.595,4.324)	0.145
Diabetes	32.675 (13.941,249.690)	0.000^*^	56.719 (12.712,134.213)	0.000^*^	19.514 (5.246,72.348)	0.000^*^
GERD	13.342 (2.562,114.103)	0.003^*^	0.814 (0.792,14.103)	0.002^*^	0.812 (0.693,1.562)	0.558
Hypercholesterolemia	0.456 (0.231,2.379)	0.372	0.923 (0.686,2.147)	0.348	14.094 (3.527,94.232)	0.003^*^
Hypertriglyceridemia	0.789 (0.546,1.456)	0.577	0.581 (0.372,2.279)	0.691	3.799 (1.272,15.410)	0.002^*^
Fatty liver	23.128 (10.905,287.514)	0.001^*^	13.762 (9.413,65.792)	0.003^*^	0.913 (0.825,2.018)	0.164

*GERD, gastroesophageal reflux disease; MAU, microalbuminuria; 25 (OH) D, 25 hydroxyvitamin D; PTH, parathyroid hormone*.

* *P <0.05*.

## Discussion

In this study, the most prominent nutritional deficiencies in these candidates for bariatric surgery were vitamin D, globulin, albumin, zinc, sodium, folate, and chloride. Elevated PTH levels and MAU were also noted in these populations. Serum ferritin deficit appeared more prevalent in women than men. This difference can be attributed to the ongoing menstrual blood loss that only occurs in female (14). Therefore, sufficient attention should be paid to the existing electrolyte/nutrient deficiencies of bariatric candidates. What has caught our attention was that in the electrolyte/nutrient test, some indicators, such as vitamins, MAU, PTH, ferritin, etc., have a small sample size, which may have a certain impact on the results obtained, and a larger sample size was needed for further research.

Vitamin D deficiency was the most common deficiency in the Chinese candidates. Recent research also reported vitamin D deficiencies in 60–93% of patients before bariatric surgery (15, 16). Insufficient vitamin D is associated with a greater risk of infection, autoimmunity, cancer, insulin secretion and insulin resistance, metabolic bone disease, and osteoporosis. Our data indicated that patients with higher BMI were more prone to vitamin D deficiency, which is similar to previous studies (17–19). A study from China reported no correlation between vitamin D deficiency and BMI (20). No current research can explain the relationship between D and obesity. However, vitamin D deficiency did not exhibit a correlation with gender, age, or WC in our study. Possible explanations of vitamin D deficiency are related to reduced milk consumption, reduced sun exposure, and decreased bioavailability of vitamin D due to its accumulation in adipose tissue (21). Preoperative vitamin D deficiency is likely to remain postoperatively (22). Vitamin D supplementation is a safe and effective method to increase serum concentrations. Optimal supplementation and dosing of vitamin D has not been clearly reported in the literature and is confounded by lifestyle factors, including geographic location, season, and daily sun exposure (22, 23).

PTH is an important regulator of vitamin D, calcium homeostasis, and bone metabolism (24). Low serum calcium is registered by calcium-sensing receptors on parathyroid cells, which stimulate parathyroid glands to release PTH into the circulation (25). Elevated PTH levels were identified in 11.9% of the patients, and this level is lower than values in previous reports (15, 16, 19, 25–27), which are as high as 93% (26). Hypocalcemia was noted in 4.05% of the candidates, while other studies reported prevalence rates ranging from 0.9 to 13.7% (17, 28). One possible reason is that increased PTH levels adjust serum calcium levels, allowing more calcium to be transferred into serum from bone. Of note, not all those with vitamin D deficiency had increased PTH, which may suggest regulation by unknown factors or that current laboratory normal range for PTH is overestimated for patients with obesity. At present, there is no clear conclusion that high levels of PTH are related to obesity or are regulated by vitamin D deficiency and serum calcium. Some studies found that PTH levels remained positively associated with BMI (19, 29). Nevertheless, whether PTH is an independent predictive factor in the development of obesity or would be mediated by 25 (OH) D is still a debate.

MAU is associated with an increased risk for future cardiovascular disease, renal disease, atherosclerosis, and cardiovascular disease mortality and all-cause mortality (30). Obesity has been implicated as an independent risk factor for the development of worsening microalbuminuria and chronic kidney disease, and its progression to end-stage kidney disease. Atta et al.'s study proved that MAU significantly increased in the obese group compared with non-obese group (31). MAU was found in 44.96% of our patients, which was higher than 20.07% of the obese Indian population (32). Our study found a relationship between MAU and gender in 59.65% of men and 33.33% of women (*P* = 0.003), while other studies showed a lower prevalence (32–34). Several studies suggested that MAU exhibited a high prevalence among men with visceral obesity but not among women (35, 36). A metabolic protective effect of adiponectin is a potential reason (37). In addition, MAU tends to increase as WC increases. WC has been recognized as a more sensitive index than BMI to assess metabolic syndrome in Asian populations because Asian people are more likely to encounter central obesity (38). Although, the prevalence rates increase with increasing BMI, the result was not statistically significant (*P* = 0.105), which is similar results reported in several studies (32, 34). Tapp et al. found in their study that the prevalence of microalbuminuria increased with increasing HbA1c levels significantly (*p* = 0.001) (39). In the study published by Bagasrawala et al. hypertension and T2DM proved to be the most significant independent factors responsible for increase of microalbuminuria (32).

Few studies have reported the relationship between obesity and electrolytes. Our data indicated that the prevalence of electrolyte deficiencies was 11.02% for sodium, 7.69% for chloride, 4.05% for calcium, and 1.62% for potassium, and these values were less than those reported in other studies from China (20). We observed significant associations between gender/WC and sodium status (*P* <0.05) but not chloride and potassium. Previous studies have confirmed that even mild electrolyte disturbances are associated with adverse outcomes. Zinc plays a role in the immune system, wound healing, and synthesis of insulin. Deficiencies in serum levels of the mineral zinc were identified in a small amount of patients preoperatively, and the prevalence level was increased compared with that reported in other studies (26, 40). van Rutte et al. (41) reported normal zinc levels in all patients preoperatively.

## Conclusion

The main finding of the study is that there are many nutrient deficiencies in patients prior to bariatric surgery in South China. In obese patients before bariatric surgery, there were gender differences in globulin, sodium, microalbuminuria; Age group analyses revealed that older people (≥50) were more likely to exhibit albumin, globulin, and zinc deficiency. The albumin deficiency rate was highest in the BMI ≥ 47.5 kg/m^2^ group, and the 25 (OH) D deficiency rate was highest in the 37.5–42.5 kg/m^2^ BMI group. In terms of waist circumference classifications, participants with higher WC (≥130 cm) had higher rates of deficiencies for albumin and sodium. Limitations of the study highlighted were that for a 5-year period there was a relatively small sample size and biochemical tests were not available for all subjects. The strength was that the investigations included a relatively big number of tests with a clinical significance which is important for such a group of people.

## Data Availability Statement

The datasets used and/or analyzed during the current study are available from the corresponding author on reasonable request.

## Ethics Statement

Ethical review and approval was not required for the study on human participants in accordance with the local legislation and institutional requirements. Written informed consent for participation was not required for this study in accordance with the national legislation and the institutional requirements.

## Author Contributions

WS and ChunxW collected preoperative trace element data for patients undergoing bariatric surgery. ChunjW and LS analyzed these data and was the main contributor in writing the manuscript. LS, WS, and ChunxW reviewed and revised the manuscript. The final manuscript read and approved by all authors.

## Conflict of Interest

The authors declare that the research was conducted in the absence of any commercial or financial relationships that could be construed as a potential conflict of interest.

## References

[1] Haslam DW James WP. Obesity. Lancet. (2005) 366:1197–209. 10.1016/S0140-6736(05)67483-1 16198769

[2] Li Y Teng D Shi X Qin G Qin Y Quan H. Prevalence of diabetes recorded in mainland china using 2018 diagnostic criteria from the American Diabetes Association: National Cross Sectional Study. BMJ. (2020) 369:m997. 10.1136/bmj.m997 32345662 PMC7186854

[3] Tremmel M Gerdtham UG Nilsson PM Saha S. Economic burden of obesity: a systematic literature review. Int J Environ Res Public Health. (2017) 14:435. 10.3390/ijerph14040435 28422077 PMC5409636

[4] GBD 2017 Risk Factor Collaborators. Global, regional, and national comparative risk assessment of 84 behavioural, environmental and occupational, and metabolic risks or clusters of risks for 195 countries and territories, 1990-2017: a systematic analysis for the Global Burden of Disease Study 2017. Lancet. (2018) 392:1923–94. 10.1016/S0140-6736(18)32225-6 30496105 PMC6227755

[5] Longitudinal Assessment of Bariatric Surgery (LABS) Consortium Flum DR Belle SH King WC Wahed AS Berk P. Perioperative safety in the longitudinal assessment of bariatric surgery. N Engl J Med. (2009) 361:445–54. 10.1056/NEJMoa0901836 19641201 PMC2854565

[6] Gong K Gagner M Pomp A Almahmeed T Bardaro SJ. Micro-nutrient deficiencies after laparoscopic gastric bypass: recommendations. Obes Surg. (2008) 18:1062–6. 10.1007/s11695-008-9577-9 18535863

[7] Gillon S Jeanes YM Andersen JR Våge V. Micronutrient status in morbidly obese patients prior to laparoscopic sleeve gastrectomy and micronutrient changes 5 years post-surgery. Obes Surg. (2017) 27:606–12. 10.1007/s11695-016-2313-y 27491294

[8] Nicoletti CF Lima TP Donadelli SP Salgado W Jr Marchini JS Nonino CB. New look at nutritional care for obese patient candidates for bariatric surgery. Surg Obes Relat Dis. (2013) 9:520–5. 10.1016/j.soard.2011.08.010 21978750

[9] Damms-Machado A Friedrich A Kramer KM Stingel K Meile T Küper MA. Pre- and postoperative nutritional deficiencies in obese patients undergoing laparoscopic sleeve gastrectomy. Obes Surg. (2012) 22:881–9. 10.1007/s11695-012-0609-0 22403000

[10] Ben-Porat T Elazary R Yuval JB Wieder A Khalaileh A Weiss R. Nutritional deficiencies after sleeve gastrectomy: can they be predicted preoperatively? Surg Obes Relat Dis. (2015) 11:1029–36. 10.1016/j.soard.2015.02.018 25857443

[11] Parrott J Frank L Rabena R Craggs-Dino L Isom KA Greiman L. American Society for metabolic and bariatric surgery integrated health nutritional guidelines for the surgical weight loss patient 2016 update: micronutrients. Surg Obes Relat Dis. (2017) 13:727–41. 10.1016/j.soard.2016.12.018 28392254

[12] Caspard H Jabbour S Hammar N Fenici P Sheehan JJ Kosiborod M. Recent trends in the prevalence of type 2 diabetes and the association with abdominal obesity lead to growing health disparities in the USA: an analysis of the NHANES surveys from 1999 to 2014. Diabetes Obes Metab. (2018) 20:667–71. 10.1111/dom.13143 29077244 PMC5836923

[13] Wang H Wang J Liu MM Wang D Liu YQ Zhao Y. Epidemiology of general obesity, abdominal obesity and related risk factors in urban adults from 33 communities of Northeast China: the CHPSNE study. BMC Public Health. (2012) 12:967. 10.1186/1471-2458-12-967 23146089 PMC3509037

[14] Ross EM. Evaluation and treatment of iron deficiency in adults. Nutr Clin Care. (2002) 5:220–4. 10.1046/j.1523-5408.2002.05503.x 12455223

[15] Carlin AM Rao DS Meslemani AM Genaw JA Parikh NJ Levy S. Prevalence of vitamin D depletion among morbidly obese patients seeking gastric bypass surgery. Surg Obesity Relat Dis. (2006) 2:98–103. 10.1016/j.soard.2005.12.001 16925330

[16] Peterson LA Cheskin LJ Furtado M Papas K Schweitzer MA Magnuson TH. Malnutrition in bariatric surgery candidates: multiple micronutrient deficiencies prior to surgery. Obes Surg. (2016) 26:833–8. 10.1007/s11695-015-1844-y 26297429

[17] Krzizek EC Brix JM Herz CT Kopp HP Schernthaner GH Schernthaner G. Prevalence of micronutrient deficiency in patients with morbid obesity before bariatric surgery. Obes Surg. (2018) 28:643–8. 10.1007/s11695-017-2902-4 28849358

[18] Moizé V Deulofeu R Torres F de Osaba JM Vidal J. Nutritional intake and prevalence of nutritional deficiencies prior to surgery in a Spanish morbidly obese population. Obes Surg. (2011) 21:1382–8. 10.1007/s11695-011-0360-y 21298509

[19] Censani M Stein EM Shane E Oberfield SE McMahon DJ Lerner S. Vitamin D deficiency is prevalent in morbidly obese adolescents prior to bariatric surgery. ISRN Obes. (2013) 2013:284516. 10.1155/2013/284516 23724340 PMC3664934

[20] Wang C Guan B Yang W Yang J Cao G Lee S. Prevalence of electrolyte and nutritional deficiencies in Chinese bariatric surgery candidates. Surg Obes Relat Dis. (2016) 12:629–34. 10.1016/j.soard.2015.12.009 27012874

[21] Wortsman J Matsuoka LY Chen TC Lu Z Holick MF. Decreased bioavailability of vitamin D in obesity. Am J Clin Nutr. (2000) 72:690–3. 10.1093/ajcn/72.3.690 10966885

[22] Peterson LA Zeng X Caufield-Noll CP Schweitzer MA Magnuson TH Steele KE. Vitamin D status and supplementation before and after bariatric surgery: a comprehensive literature review. Surg Obes Relat Dis. (2016) 12:693–702. 10.1016/j.soard.2016.01.001 27036669

[23] Kasahara AK Singh RJ Noymer A. Vitamin D (25OHD) serum seasonality in the United States. PLoS ONE. (2013) 8:e65785. 10.1371/journal.pone.0065785 23805188 PMC3689782

[24] Khundmiri SJ Murray RD Lederer E. PTH and vitamin D. Compr Physiol. (2016) 6:561–601. 10.1002/cphy.c140071 27065162 PMC11163478

[25] Krishnan V Ma YL Chen CZ Thorne N Bullock H Tawa G. Repurposing a novel parathyroid hormone analogue to treat hypoparathyroidism. Br J Pharmacol. (2018) 175:262–27. 10.1111/bph.14028 28898923 PMC5758387

[26] Sánchez A Rojas P Basfi-Fer K Carrasco F. Micronutrient deficiencies in morbidly obese women prior to bariatric surgery. Obes Surg. (2016) 26:361–8. 10.1007/s11695-015-1773-9 26108638

[27] Schweiger C Weiss R Berry E Keidar A. Nutritional deficiencies in bariatric surgery candidates. Obes Surg. (2010) 20:193–7. 10.1007/s11695-009-0008-3 19876694

[28] Toh SY Zarshenas N Jorgensen J. Prevalence of nutrient deficiencies in bariatric patients. Nutrition. (2009) 25:1150–6. 10.1016/j.nut.2009.03.012 19487104

[29] Snijder MB van Dam RM Visser M Deeg DJ Dekker JM Bouter LM. Adiposity in relation to vitamin D status and parathyroid hormone levels: a population-based study in older men and women. J Clin Endocrinol Metab. (2005) 90:4119–23. 10.1210/jc.2005-0216 15855256

[30] Yuyun MF Khaw KT Luben R Welch A Bingham S Day NE. Microalbuminuria independently predicts all-cause and cardiovascular mortality in a British population: the European Prospective Investigation into Cancer in Norfolk (EPIC-Norfolk) population study. Int J Epidemiol. (2004) 33:189–98. 10.1093/ije/dyh008 15075168

[31] Atta M Abdalla N Ibrahim A. Microalbuminuria and adiponectin in obese nondiabetic nonhypertensive people. Egypt J Obes Diabetes Endocrinol. (2016) 2:156–62. 10.4103/2356-8062.200938

[32] Bagasrawala SI Sheth H Shah H Ansari R Lakdawala M. Metabolic syndrome rather than obesity alone is more significant for kidney disease. Obesity Surg. (2019) 29:3478–83. 10.1007/s11695-019-04011-2 31190265

[33] Chen F Yang W Weng J Jia W Ji L Xiao J. Albuminuria: prevalence, associated risk factors and relationship with cardiovascular disease. J Diabetes Investig. (2014) 5:464–71. 10.1111/jdi.12172 25411608 PMC4210073

[34] Afkhami-Ardekani M Modarresi M Amirchaghmaghi E. Prevalence of microalbuminuria and its risk factors in type 2 diabetic patients. Indian J Nephrol. (2008) 18:112–7. 10.4103/0971-4065.43690 20142916 PMC2813138

[35] Kim H Kim HJ Shin N Han M Park H Kim M. Visceral obesity is associated with microalbuminuria in nondiabetic Asians. Hypertens Res. (2014) 37:679–84. 10.1038/hr.2014.47 24646640

[36] Foster MC Hwang SJ Massaro JM Hoffmann U DeBoer IH Robins SJ. Association of subcutaneous and visceral adiposity with albuminuria: the Framingham Heart Study. Obesity. (2011) 19:1284–9. 10.1038/oby.2010.308 21183930 PMC3096746

[37] He H Ni Y Chen J Zhao Z Zhong J Liu D. Sex difference in cardiometabolic risk profile and adiponectin expression in subjects with visceral fat obesity. Transl Res. (2010) 155:71–7. 10.1016/j.trsl.2009.08.003 20129487

[38] Kasama K Mui W Lee WJ Lakdawala M Naitoh T Seki Y. IFSO-APC consensus statements2011. Obes Surg. (2012) 22:677–84. 10.1007/s11695-012-0610-7 22367008

[39] Tapp RJ Shaw JE Zimmet PZ Balkau B Chadban SJ Tonkin AM. Albuminuria is evident in the early stages of diabetes onset: results from the Australian Diabetes, Obesity, and Lifestyle Study (AusDiab). Am J Kidney Dis. (2004) 44:792–8. 10.1016/S0272-6386(04)01079-0 15492944

[40] Lefebvre P Letois F Sultan A Nocca D Mura T Galtier F. Nutrient deficiencies in patients with obesity considering bariatric surgery: a cross-sectional study. Surg Obes Rel Dis. (2014) 10:540–6. 10.1016/j.soard.2013.10.003 24630922

[41] van Rutte PW Aarts EO Smulders JF Nienhuijs SW. Nutrient deficiencies before and after sleeve gastrectomy. Obes Surg. (2014) 24:1639–46. 10.1007/s11695-014-1225-y 24706197

